# Congenital Pharyngeal Web in an Adult: Treatment of a Rare Clinical Anomaly by Coblation

**DOI:** 10.51894/001c.12473

**Published:** 2020-06-08

**Authors:** Katrina Minutello, Steven Pinther, Robert Stachler

**Affiliations:** 1 OMS III MSUCOM; 2 Otolaryngology Ascension Macomb-Oakland; 3 Stachler ENT

**Keywords:** dysphagia, pharyngeal web, coblation, hypopharynx

## Abstract

**INTRODUCTION TO THE TOPIC:**

Previous reports of congenital pharyngeal webs, although rare, have been described in children. Clinical presentation varies, ranging from aspiration to intermittent airway obstruction, and most commonly, dysphagia. In this case report, the authors describe an unusual finding of a hypopharyngeal web in an adult patient. This patient had no prior history of chemoradiotherapy, malignancy, or total laryngectomy, all of which have been associated with acquired pharyngeal stenosis, supporting that this finding was of congenital origin. After a review of the possible embryological developmental abnormalities, the hypothesis is that of gut recanalization failure during development.

**CASE PRESENTATION:**

We present a case of a woman in her mid-40’s with a history of solid food dysphagia resulting in a 20 kg weight loss over three months. The patient denied dysphagia progressing to liquids, pain with swallowing, and a history of alcohol or tobacco use. Upon examination of the larynx via laryngoscope, a congenital hypopharyngeal web was identified. Successful excision of the web via coblation restored proper drainage of the pyriform sinus into the esophagus and resulted in markedly improved swallowing function and weight gain.

**CONCLUSIONS:**

Pharyngeal webs are rare findings, particularly in adult patients. These congenital anomalies can be safely and effectively treated endoscopically via coblation.

## INTRODUCTION

Pharyngeal webs are rare congenital anomalies characterized by mucosal bands extending from the posterior pharyngeal wall anteriorly to the glottis, which is the opening between the vocal cords.^1^ This type of defect in laryngeal anatomy is an uncommon occurrence, particularly in the adult population. Subsequent to the first case reported in 1983 by Gerson et al., six cases of congenital pharyngeal webs have been described in the pediatric population with mild variation in their clinical presentation and management.^1–4^ In adults, congenital webs must be differentiated from hypopharyngeal stenosis, an acquired complication in the lower pharynx secondary to iatrogenic trauma, ingestion of hazardous substances, chemoradiation, or malignancy.^5–10^

A mucosal band located in the hypopharynx, the most inferior portion of the pharynx, obstructs the normal passage of a food bolus from the oral cavity through the opening of the esophagus. As a result, patients have presented with intermittent airway obstruction, aspiration, and odynophagia (pain with swallowing).^1–4^ Most commonly, patients with webs in the hypopharynx present with progressive solid food dysphagia (i.e., difficulty swallowing).^1^ This case demonstrates how congenital anomalies must be considered in the workup of dysphagia, even in adult patients. A thorough evaluation of the oral cavity and larynx is necessary for diagnosis and exclusion of non-anatomical causes of origin.

In this paper, we report the second reported case of a congenital hypopharyngeal web in an adult patient and describe the potential functional and clinical implications of this anomaly. Additionally, we will review the differential diagnoses for primary care providers and otolaryngologists alike. Previous cases have described the exclusive use of carbon dioxide (CO2) laser in the removal of pharyngeal webs. Controlled ablation, or coblation, was originally introduced in the late 1990s for arthroscopic surgeries. This minimally invasive modality involves the use of radiofrequency at a low temperature to precisely remove tissue and since its introduction, has proved ideal for use in a wide range of otolaryngologic procedures. This includes, but is not limited to: adenotonsillectomy, tongue base reduction, removal of laryngeal papilloma, and nasal polypectomy.^11^ To the authors’ knowledge, this is the first known report of congenital pharyngeal web excision via coblation.

## CASE REPORT

A woman in her mid-40’s with past medical history significant for Crohn’s disease, anemia, and esophagitis (i.e., inflammation of the esophagus) presented with a 20 kg weight loss secondary to worsening solid food dysphagia during the past three months. She denied difficulty initiating a swallow, dysphagia progressing to liquids, painful swallowing, nausea or vomiting and reported no fevers. The patient had no history of smoking or alcohol use. She reported having similar symptoms of dysphagia over many years tolerated with diet modification and several uncomplicated balloon esophagoscopies during the past three years with temporary symptomatic relief. Initial physical exam findings were unremarkable.

**Figure 1:**
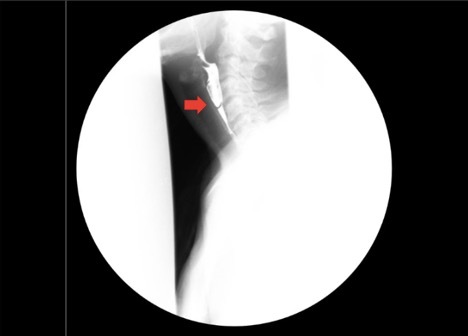
XR esophagram with barium swallow demonstrating an incomplete, anterior cricopharygeal esophageal web at the level of C5-C6

Based on the patient’s presenting symptoms, a barium swallow (i.e., upper gastrointestinal tract x-ray exam) was obtained, which demonstrated an incomplete cricopharyngeal web (i.e., mucosal web that tethers the lateral hypopharyngeal wall to the cricopharyngeus muscle) (red arrow in Figure 1), extending from the anterior wall at the level of C5-C6 with barium passing through the web without appreciable delay.

**Figure 2:**
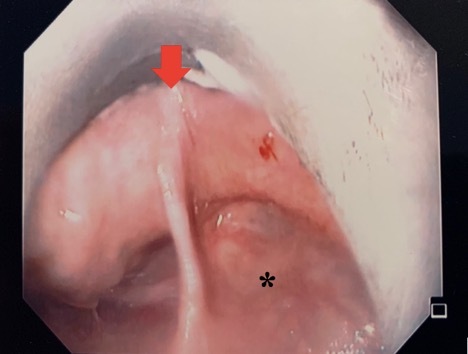
Close-up showing midline pharyngeal web extending from posterior pharynx wall anteriorly to the posterior cricoid, separating the right pyriform sinus* from the esophageal introitus

Two days following imaging, the patient was taken to the operating room for esophagoscopy with dilation and micro suspension direct laryngoscopy. The patient was instructed to withhold food and fluids 8 hours prior to her procedure. The laryngoscope revealed no anatomical abnormalities within the larynx or trachea. Within the hypopharynx (most inferior portion of the pharynx), a large midline web (red arrow in Figure 2) extended from the posterior cricoid area (region of the throat just behind the cricoid cartilage) and right pyriform sinus (subsite of the hypopharynx that act as a lateral channel for the passage of boluses into the esophagus) to the posterior pharyngeal wall, essentially separating the right pyriform sinus from the remainder of the pharyngeal inlet.

This was located at 16 cm from the incisors with no overlying ulceration. The esophagus was dilated over a wire without complications.

**Figure 3:**
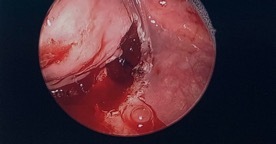
Immediate postoperative view after removal of the pharyngeal web via coblation

Two months later, the patient underwent suspension microlaryngoscopy (direct visualization of the larynx via laryngoscope) and esophageal dilation over wire, which is the careful widening of a stenosed, or narrowed, section of the esophagus wall. The web was excised and the pharyngeal tissue was biopsied. Using coblation technique, the authors achieved clean excision of the mucosal tissue without damage to surrounding structures, allowing the pyriform sinus to drain into the esophagus. (Figure 3)

The esophagus was again dilated over wire without complications. Histopathologic analysis of the pharyngeal web tissue reported hyperplastic mildly atypical squamous mucosa, and a lack of abnormal cell types suggestive of a malignant process (i.e., dysplasia). The immunohistochemical stains performed on the cells were negative for p16, a tumor suppressor protein highly correlated with human papilloma virus (HPV) infection in squamous cell carcinoma of the head and neck. This further supported the tissue was not malignant in nature.

Postoperatively, the patient was advised to begin with a clear liquid diet and advance to solid foods as tolerated. By the beginning of the second week postoperatively, she reported significantly improved swallowing function and tolerated a solid food diet without dysphagia or complications. She has been steadily gaining weight.

## DISCUSSION

Pharyngeal webs are extremely rare congenital anomalies characterized by a mucosal band-like extension from the posterior pharyngeal wall anteriorly to the glottis.^1^ This condition was first reported by Gerson et al. in 1983 and has been subsequently described in five other pediatric cases, in which the majority of children had concurrent syndromic diagnoses or webs involving the larynx.^1–4^

Articles that reported pharyngeal webs in adults described hypopharyngeal stenosis secondary to radiation, squamous cell carcinoma, total laryngectomy, hemipharyngeal flap reconstruction, or blistering diseases.^5,7^ Prior to this case, bilateral hypopharyngeal webs have only been described once in an adult female with a medical history only significant for gastroesophageal reflux disease (GERD).^8^ To the authors’ knowledge, this is the second documented case of a congenital hypopharyngeal web found in an adult patient.

Differentiation of congenital pharyngeal webs from acquired pharyngeal stenosis or esophageal webs is necessary for appropriate prevention, treatment and management. As described in this case, congenital webs are non-ulcerated, non-cicatricial band-like extensions with smooth overlying mucosa extending from the posterior pharyngeal wall anteriorly.^1^ Pharyngeal synechiae, acquired scar tissue formation resulting in adhesion between two mucosal surfaces, has been associated with blistering diseases such as toxic epidermal necrolysis (Lyell syndrome) or epidermolysis bullosa following mucosal damage.^5^ Congenital pharyngeal webs must also be differentiated from pharyngeal stenosis, a complication resulting from total laryngectomy, ingestion of hazardous substances, chemoradiotherapy, or malignancy of the pyriform sinus.^5–10^ Unlike pharyngeal webs, esophageal webs (as seen in Plummer-Vinson syndrome) are located more distally to the cricopharyngeal muscle.

This patient had a history of several uncomplicated esophagoscopies with an endoscopic procedure to widen regions of narrowed lumen in the esophagus (i.e., balloon dilation) performed. Previously reported complications of this procedure, although rare, include esophageal perforation, intramural laceration, infection, and acute esophagitis secondary to an increased risk for GERD.^12^ Rather, pharyngeal stenosis resulting from iatrogenic trauma has been associated with other otolaryngologic surgical procedures such as total laryngectomy, adenoidectomy and tonsillectomy, and pharyngectomy.^13^

Prior studies have suggested that congenital pharyngeal webs arise due to a failure in the development of brachial arches.^3^ Two cases reported pharyngeal webs extending from the posterior pharyngeal wall to the base of the tongue associated with absent faucial pillars (the anterior and posterior boarders of the tonsillar fossa) and tonsils on the affected side, suggesting the failure of the second pharyngeal pouch.^3,4^ Another study reported cases of pharyngeal webs located more inferiorly and associated with laryngo-tracheal anomalies, suggesting the malformations arose from failure in development from the 4^th^ and/or 6^th^ arches.^1^ In this case report, the patient did not have any associated developmental anomalies. We have concluded that isolated failure of the patient’s foregut recanalization during embryonic development gives rise to her congenital pharyngeal webs.

Clinical presentation of hypopharyngeal webs vary, ranging from aspiration to intermittent airway obstruction.^2^ Most commonly, patients present with solid food dysphagia.^1,4,5^ Dysphagia is a common issue, affecting 7-10% of patients over 50-years-old in all healthcare settings.^14^ The provider typically confirms the diagnosis with a thorough history and physical exam, barium swallow, and/or gastroesophageal endoscopy. In the event the etiology of dysphagia is unclear or further diagnostic testing is necessary, referral to subspecialists is warranted.

Laryngoscopy, performed by otolaryngologists, is necessary to confirm the diagnosis and potentially identify associated pharyngolaryngeal anomalies. The medical literature that has made mention of web excision techniques has described suspension laryngoscopy with CO2 laser and suturing as an effective method.^1,4^ In patients who are unresponsive to more conservative surgical intervention, local mucosal flaps have been necessary.^7^ Coblation, which involves radiofrequency at low temperatures and saline solution, is a commonly used modality in otolaryngologic surgeries in both adults and children due to the low risk of injuring surrounding tissue.^11^ While coblation has been readily used in tonsillectomy, turbinate reduction, and tongue reduction surgery, this is the first reported case of the use of coblation in the removal of a congenital pharyngeal web.

## CONCLUSIONS

We report the clinical impact of hypopharyngeal webs, a rare congenital malformation, and propose the use of coblation as an effective removal technique. Due to the low incidence of presentation, particularly in adult patients, continued reporting of such anomalies are necessary to improve diagnosis, treatment, and management in the adult population.

### Conflict of Interest

The authors declare no conflict of interest.
